# Optimization of Dieldrin Selection for the Genetic Sexing of *Aedes albopictus*

**DOI:** 10.3390/insects14070630

**Published:** 2023-07-13

**Authors:** Sarah Scussel, Benjamin Gaudillat, Jérémy Esnault, Quentin Lejarre, Marianne Duployer, Daouia Messaoudi, Patrick Mavingui, Pablo Tortosa, Julien Cattel

**Affiliations:** 1 Groupement d’Intérêt Public Cyclotron Océan Indien (CYROI), 2 rue Maxime Rivière, 97490 Ste Clotilde, France; s.scussel@cyroi.fr (S.S.); b.gaudillat@cyroi.fr (B.G.); j.esnault@cyroi.fr (J.E.); m.duployer@cyroi.fr (M.D.); 2 Symbiosis Technologies for Insect Control (SymbioTIC), Plateforme de Recherche Cyroi, 2 rue Maxime Rivière, 97490 Ste Clotilde, France; quentinlejarre.symbiotic@gmail.com; 3 Groupe Berkem, 20 rue Jean Duvert, 33290 Blanquefort, France; daouia.messaoudi@berkem.com; 4 Unité Mixte de Recherche Processus Infectieux en Milieu Insulaire Tropical (UMR PIMIT), CNRS 9192, INSERM 1187, IRD 249, Université de La Réunion, Plateforme de recherché CYROI, 97490 Ste Clotilde, France; patrick.mavingui@univ-reunion.fr (P.M.); pablo.tortosa@univ-reunion.fr (P.T.)

**Keywords:** *Aedes albopictus*, genetic sexing strain, insecticide selection, dieldrin quantification

## Abstract

**Simple Summary:**

The development of sterile-male programs for the control of mosquito populations faces a number of challenges including sex separation. Genetic sexing strategies offer the advantage of limiting costs and space by removing females at the larval stage. We recently developed a genetic sexing strain in *Aedes albopictus* conferring dieldrin resistance in males only. We performed several experiments in order to reduce the quantity of dieldrin used while maintaining a high level of female elimination and recovery of nearly all resistant males. Interestingly, we showed that the use of this reduced dieldrin exposure led to a dieldrin detection in adult males that was below the sensitivity threshold of the Gas Chromatography-Mass Spectrometry detection method. The utilization of this genetic sexing strain in mosquito control programs implemented at industrial scales is discussed.

**Abstract:**

The mass production of mosquitoes at an industrial scale requires efficient sex separation, which can be achieved through mechanical, genetic or artificial intelligence means. Compared with other methods, the genetic sexing approach offers the advantage of limiting costs and space by removing females at the larval stage. We recently developed a Genetic Sexing Strain (GSS) in *Aedes albopictus* based on the sex linkage of the *rdl^R^* allele, conferring resistance to dieldrin, to the male (M) locus. It has been previously reported that dieldrin ingested by larvae can be detected in adults and bioaccumulated in predators, raising the question of its use at a large scale. In this context, we performed several experiments aiming at optimizing dieldrin selection by decreasing both dieldrin concentration and exposure time while maintaining a stable percentage of contaminating females averaging 1%. We showed that the previously used dieldrin exposure induced an important toxicity as it killed 60% of resistant males at the larval stage. We lowered this toxicity by reducing the dose and/or the exposure time to recover nearly all resistant males. We then quantified the residues of dieldrin in resistant male adults and showed that dieldrin toxicity in larvae was positively correlated with dieldrin concentrations detected in adults. Interestingly, we showed that the use of reduced dieldrin exposure led to a dieldrin quantification in adult males that was below the quantity threshold of the Gas Chromatography-Mass Spectrometry detection method. Presented data show that dieldrin exposure can be adjusted to suppress toxicity in males while achieving efficient sexing and lowering the levels of dieldrin residues in adults to barely quantifiable levels.

## 1. Introduction

Mosquito-borne diseases are among the leading causes of mortality and morbidity in humans [1]. In the absence of effective vaccines, vector control is one of the few available strategies for limiting pathogen transmission. Although the use of chemical products remains the most common method, alternatives have emerged aiming at reducing the environmental impact of vector control. Among the methods under development, the control of vector populations through the mass release of sterilizing males is appealing as it is highly specific and not polluting, and does not require the introduction of any new species into the environment [2,3]. The Sterile Insect Technique (SIT) based on the release of sterile male insects that search and mate with wild females to prevent subsequent production of offspring has been successfully used to control various insect pest species [4]. Closely related to SIT, the Incompatible Insect Technique (IIT) is based on the massive release of *Wolbachia*-infected males that induce sterility in wild-type females, [2,5]. IIT, SIT or their combination require a robust sex separation system, especially in mosquitoes, because the accidental release of females creates a biting nuisance and may have detrimental epidemiological consequences.

In mosquitoes, a variety of sex separation methods have been established and are based on size and developmental dimorphisms, morphological or behavioral traits [6]. Since none of these methods are perfect, most large scale implementation of sterile-male programs use as a complement either irradiation or artificial intelligence to prevent risks associated with accidental releases of fertile females. Although irradiation and artificial intelligence approaches have demonstrated their use in area-wide programs aiming at controlling mosquito populations [7,8,9], both increase the cost of such programs [10]. Genetic approaches have been proposed as an alternative to quickly separate large quantities of males and females at an early development stage, thus reducing the time and cost of production [11]. For example, a Genetic Sexing Strain (GSS) has been developed for the mosquito *Anopheles stephensi* based on the expression of a green fluorescent protein at the third instar larvae stage in males only [12,13]. Recently, an *Aedes albopictus* transgenic line was constructed in which *Nix* is expressed ectopically, inducing masculinization in combination with the expression of a fluorescent marker [14]. Other more classical genetic approaches have been developed aiming at randomly translocating (through irradiation) a conditionally lethal or selectable marker near the male-determining factor/chromosome. The majority of GSS developed through chromosomal translocations use an insecticide-resistance gene as a selectable marker, as originally developed for *Culex tarsalis* [15] and *Anopheles arabiensis* [16]. Similarly, our group recently developed an *Aedes albopictus* GSS line-based linkage of the *rdl* gene conferring resistance to dieldrin to the male locus [17] and allowing the production of 98% of males following selection at third-instar larvae [18].

It has been shown that dieldrin ingested by the larvae of an *An*. *Arabiensis* GSS (ANO IPCL1) during sex selection can be detected in adults [16]. In this context, we optimized the protocol developed by Lebon et al. [18] in order to reduce larval exposure to dieldrin. We demonstrated that the selection protocol can be easily modulated by modifying the dose and/or exposure time allowing to recover nearly all resistant males while maintaining a female contamination rate averaging 1%. We also showed that in certain conditions, dieldrin induced mortality in resistant male larvae and that this toxic effect is positively correlated with the dieldrin quantity detected in adults. Finally, and most interestingly, we showed that using optimal insecticide exposure, the dieldrin detected in adults was below the sensitivity threshold of the detection method. All these results showed that dieldrin selection can be adjusted so that sexing remains efficient while eliminating the toxicity on resistant males and maintaining the levels of dieldrin residues at hardly quantifiable levels.

## 2. Materials and Methods

### 2.1. Mosquito Strain and Rearing Conditions

All experiments were performed using the *Aedes albopictus* GSS referred to as Tikok, obtained by sex linkage of the *rdl^R^* allele, conferring dieldrin resistance [18]. The Tikok line was maintained in conditions described in Lebon et al. [18] from its original construction up to the F34 (generation used for the presented experiments). Briefly, at each generation, eggs were allowed to hatch for 24 h in a 250 mL jar to obtain approximately 1000 first instar larvae per jar. Larvae were transferred in a tray (34 × 23 × 4 cm) and fed with TetraMin (©TETRA) until the L3 stage. Larvae were then exposed to a dieldrin concentration of 0.1 ppm for 24 h. All survivors were rinsed, transferred in a tray and fed until pupation. All pupae were manually sex sorted under a microscope to determine the female contamination rate at each generation. Females were discarded and male pupae were placed in an adult cage (30 × 30 × 30 cm, Bugdorm 4S3030, Taichung, Taiwan), crossed with females from the dieldrin susceptible line (S-RUN line, see [18]) and adults were maintained under classic climatic conditions for reproduction (27 ± 1 °C, 70 ± 5% RH, 12:12 h light:dark photoperiod).

### 2.2. Dieldrin Selection at the L1 Stage

With the aim to improve the dieldrin selection protocol, we tested sexing efficiency using 1-day-old rather than 3-days-old larvae, with the same dieldrin concentration and exposure time. This protocol was used from the F34 to the F42 (generation used for the following experiments) and the female contamination rate was measured at each generation.

### 2.3. Effect of Dieldrin Concentration on Sexing

We optimized the dieldrin selection protocol performed on 1-day-old larvae by testing different dieldrin concentrations in order to decrease the quantity of used dieldrin. Six different concentrations were tested: 0.01, 0.02, 0.04, 0.06, 0.08 and 0.1 ppm. For each concentration, 1000 1-day-old larvae were manually counted, fed with TetraMin (©TETRA), and exposed to dieldrin for 24 h, then rinsed with clean water and reared until the pupal stage. Of note, 1000 1-day-old larvae were exposed in all dieldrin experiments implemented in the present investigation. The experiment was duplicated for each dose and all pupae were eventually manually sex sorted under a binocular loop to determine the female contamination rate.

### 2.4. Effect of Dieldrin Exposure Time on Sex Sorting

We tested different times of exposure at a dieldrin concentration of 0.08 ppm (the lowest concentration leading to a female contamination rate < 1% for 1000 1-day-old larvae) to reduce insecticide exposure. For this, 1-day-old larvae were exposed to dieldrin for different times: 1 h, 2 h, 4 h, 6 h, 12 h, 18 h and 24 h, then rinsed with clean water and reared until pupal stage.

### 2.5. Measurement of Dieldrin Toxicity Effect on Resistant Males

Although males are genetically resistant to dieldrin, it is important to tune insecticide exposure conditions to obtain the highest sex separation without detrimental effects on resistant males [19]. To quantify this potential toxicity, we exposed 1 day-old larvae to a dieldrin concentration of 0.08 ppm for three distinct exposure times: 0, 6 and 24 h (four replicates for each exposure time). Male recovery was used as a proxy of dieldrin toxicity and calculated as follows: male recovery (%) = [mean (number of male pupae recovered after dieldrin selection)/mean (number of male pupae recovered without selection) × 100].

### 2.6. Quantification of Dieldrin Residues in Resistant Male Adults

#### 2.6.1. Quantification at a Small Production Scale

We quantified dieldrin in adult mosquitoes after larvae selection protocols inducing either low or high toxicity on resistant male larvae. For this, 1-day-old larvae were exposed to a dieldrin concentration of 0.08 ppm for three different exposure times: 0, 6 and 24 h. Six and twenty repetitions were implemented for 6 h and 24 h exposure times, respectively, in order to obtain over 2000 surviving male pupae at each condition. For each exposure time, all surviving larvae were pooled and reared until pupation. Male recovery was calculated as described above. All male pupae were allowed to emerge in an adult cage (30 × 30 × 30 cm, Bugdorm 4S3030) and fed with a 5% sucrose solution. Two-four days-old adult males were frozen at −20 °C for 1 h and for each selection time, three 50 mL vials containing 500 adult males were used for dieldrin quantification.

#### 2.6.2. Quantification of Dieldrin in a Mass Production Context

After quantifying dieldrin residues in adults exposed at different times, we repeated quantification in a mass production context. For this, brushed eggs (160 mg) were allowed to hatch for 8 h in a 200 mL pot containing 30 mL of hatching solution (tap water at 5% of TetraMin (©TETRA)). One-day-old larvae were then exposed for 16 h to five different dieldrin concentrations: 0.1, 0.2, 0.4, 0.6 and 1 ppm, then rinsed with clean water and reared until pupation. All trays were supplemented with the same quantity of food (tetraMin (©TETRA), day 1: 0.3 g, day 2: 0.6 g, day 3: 20 g, day 4: 25, day 5: 20 g, day 6: 15 g, day 7: 10 g), pupae were then collected and sex separated using a pupae sex sorter (Wolbaki, WBK-P0001-V1 model). All pupae were counted using a mosquito pupae counter (Radiation General Ltd., Budapest, Hungary) [20]. Three replicates were performed and each dieldrin concentration and female contamination rate as well as male recovery were measured for each replicate. All male pupae were allowed to emerge in adult cages (30 × 30 × 30 cm, Bugdorm 4S3030) and fed with a 5% sucrose solution. Two-four days-old males were frozen at −20 °C for 1 h and 3 pools of adult males (2000 each) were frozen for subsequent dieldrin quantification for each dieldrin concentration. A female contamination rate and a male recovery rate were associated with each dieldrin concentration for each pool (corresponding to a specific tray).

### 2.7. Dieldrin Extraction and Quantification

The quantification was performed by an independent private laboratory (Groupe Berkem, Blanquefort, France). All samples were weighed before extraction. All adult males were then transferred to a vial containing 20 mL of acetonitrile (for HPLC, Fisher Chemical™, Illkirsh, France) and placed in an ultrasonic bath (Branson 1510 Ultrasonic Cleaner, Bransonic, Danbury, CT, US) at 45 °C for 90 min under agitation. The content was then filtered and transferred into a new vial until complete evaporation of acetonitrile. Two milliliters of isohexane (≥95%, for HPLC, VWR Chemicals, Rosny-sous-bois, France) were added and the quantification was performed through GS/MS/MS with a sensitivity threshold of 2 µg/L (Thermo, GC Trace 1310/TSQ 9000, Milan, Italy).

### 2.8. Statistical Analysis

Fisher exact tests were used to compare the percentage of female contamination rate (%) between protocols expositing 1-day-old or 3-day-old larvae, the female contamination rate (%) obtained after different dieldrin concentrations or exposure times, and the male recovery (%) after different dieldrin exposure times.

## 3. Results

### 3.1. Stability of Tikok Line and Efficiency of Sex Sorting at L1 Stage

With the aim to improve the dieldrin selection protocol, we tested sexing efficiency using 1 day-old rather than 3 day-old larvae. We showed that the female contamination rate was stable across generations, from the F27 to the F42, with a percentage comprised between 1.29% and 0.40% (Table 1). We did not detect differences in female contamination between protocols exposing 3-days-old (from F27 to F34) or 1-day-old larvae (from F35 to F42), with a female contamination rate averaging 0.84 (±0.26) and 0.75 (±0.17), respectively (Fisher exact test, *p* = 0.161).

### 3.2. Effect of Dieldrin Concentration on the Female Elimination Efficiency

**Figure 1:**
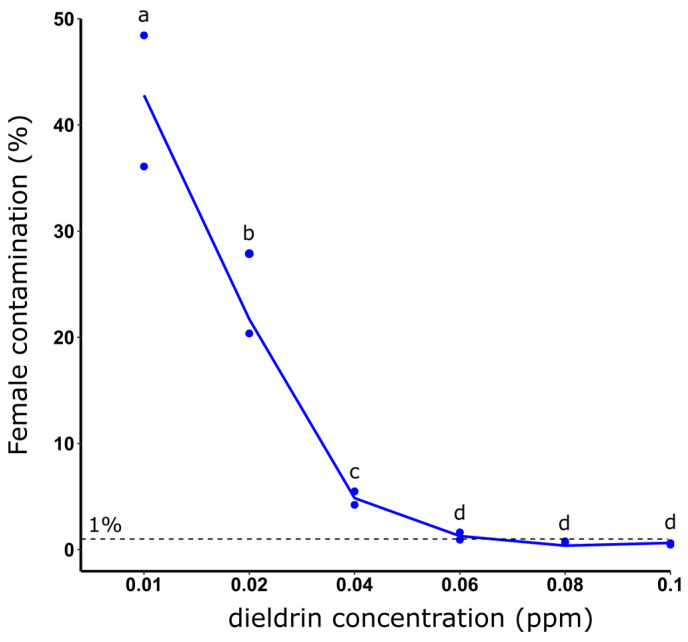
Female contamination rate (%) after 1-day-old larvae selection using different dieldrin concentrations for 24 h. Distinct letters indicate significant variations between the different dieldrin concentrations (Fisher’s exact test, *p* ≤ 0.05).

In order to lower the dieldrin quantity for sex separation, we tested different dieldrin concentrations. Increasing dieldrin exposure improved female elimination efficiency with a female contamination rate averaging 45.26% and 0.54% for dieldrin concentrations of 0.01 and 0.1 ppm, respectively (Figure 1). The female contamination rate decreased significantly for concentrations of 0.01 to 0.06 ppm and then decreased slightly until 0.1 ppm of dieldrin (Figure 1).

### 3.3. Effect of Dieldrin Exposure Time on Sex Sorting

**Figure 2:**
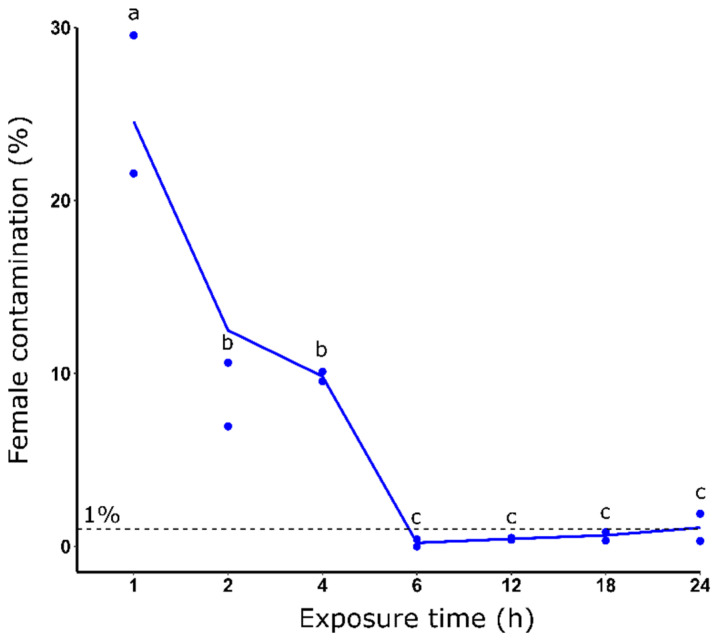
Female contamination rate (%) after dieldrin selection using different exposure times and 0.08 ppm dieldrin. Distinct letters indicate significant variations between different dieldrin concentrations (Fisher’s exact test, *p* ≤ 0.05).

We then tested different times of exposure using a constant dieldrin concentration of 0.08 ppm. Increasing exposure time increased the female elimination efficiency from 25.55% to 1.10% of the average female contamination rate, for 1 h and 24 h of exposure, respectively (Figure 2). More precisely, the female contamination rate decreased significantly from 1 to 6 h of selection. Interestingly, after 6 h and until 24 h of selection, we found a slight increase in the female mean contamination rate from 0.21% to 1.10%.

### 3.4. Measurement of Dieldrin Toxicity on Resistant Males

**Figure 3:**
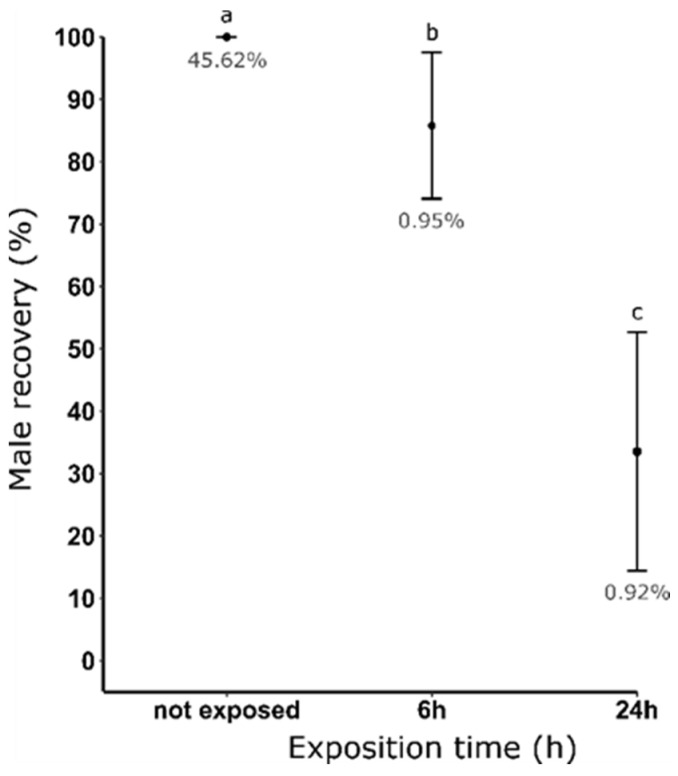
Male recovery (%) after dieldrin selection at 0.08 ppm and using different exposure times. Male recovery (%) = [mean (number of male pupae recovered after selection)/mean (number of male recovered without selection)] × 100. The average female contamination rate (%) obtained for each condition is indicated in grey. Distinct letters indicate significant variations between the different exposure times (Fisher’s Exact Test, *p* ≤ 0.05).

To quantify a potential toxic effect of dieldrin on resistant larvae, we exposed 1-day-old larvae to a constant 0.08 ppm dieldrin concentration for increasing exposure times: 0, 6 and 24 h. We showed that increasing the exposure time from 6 to 24 h while keeping dieldrin concentration constant decreased the percentage of male recovery from 85.80% to 33.52%, respectively (Figure 3). In addition, we showed that this toxic effect on resistant male larvae did not affect the female contamination rate as this rate averaged 0.9% for both exposure times.

### 3.5. Quantification of Dieldrin Residues in Resistant Male Adults

#### 3.5.1. Quantification at a Small Production Scale

Dieldrin ingested by larvae during sex selection can be detected in adults. We thus quantified dieldrin in adult mosquitoes after larvae selection inducing either low or high toxicity on resistant male larvae. Dieldrin was detected in all mosquito samples for which 1-day-old larvae had been exposed at 0.08 ppm for 6 or 24 h. For 6 h exposure, one of three quantification replicates was below the sensitivity threshold of quantification (2 µg/L), one was at the threshold, and the last replicate revealed a 2.1 µg/L dieldrin in males, corresponding to a mean of 8.32 ± 0.3 pg dieldrin per adult male. We showed that the dieldrin quantity found in resistant male adults was positively correlated with the exposure time as mosquitoes exposed for 24 h showed approximately a thousand times more dieldrin (1.94 ± 0.6 ng per mosquito, see Table 2) than those exposed for 6 h. We showed that the female contamination rate remained low (<1%) for both exposure times. Lastly, we confirmed that extended dieldrin exposure induced mortality on resistant larvae with 33.88% of male recovery for 24 h exposure, compared with 90.70% for 6 h exposure.

#### 3.5.2. Quantification of Dieldrin in Males Obtained in a Mass Production Context

We quantified dieldrin residues in a mass production context using 1-day-old larvae exposed to increasing dieldrin concentrations. Dieldrin was detected in all samples containing mosquitoes for which larvae had been exposed for 16 h at concentrations ranging from 0.1 to 1 ppm (Table 3). For 0.1, 0.2, and 0.4 ppm, dieldrin detection was below the quantification threshold (2 µg/L), corresponding to ≤2 pg of dieldrin per adult male. Two out of the three replicates using 0.6 ppm dieldrin also led to detections below the quantification threshold (≤2.33 pg of dieldrin per adult male). For the highest tested concentration (1 ppm), we found 6.66 pg dieldrin per adult male.

**Figure 4:**
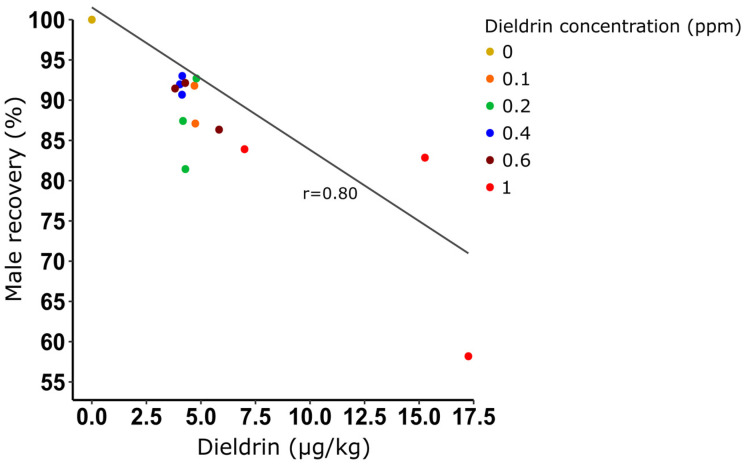
Correlation between male recovery (%) and the quantity of dieldrin residues in resistant male adults obtained in a mass production context. Male recovery (%) = [mean (number of male pupae recovered after selection)/mean (number of male recovered without selection)] × 100.

We also confirmed that exposure to a high dieldrin concentration induced mortality on resistant larvae with a male recovery of 74.97% (±14.56) for 1 ppm to be compared to lower concentrations for which the male recovery averaged 90%. Lastly, we demonstrated that dieldrin found in adults was positively correlated with male recovery (Pearson’s product-moment correlation, df = 14, *p* < 0.05) (Figure 4).

## 4. Discussion

It has been shown that dieldrin ingested by larvae during sex selection can be detected in adults [16]. It was essential to further explore this point in the context of large scale implementation of SIT or IIT programs using such a GSS. We performed several experiments aiming at reducing larval exposure to dieldrin. This optimization included a decrease in dieldrin concentration and in exposure time in order to minimize the quantity of dieldrin detected in resistant male adults.

The *Ae*. *albopictus* GSS line used in this study has been genetically stable since its construction and until the F42, corresponding to the last generation used in this study, with a female contamination rate ranging between 0.4% and 1.7%. Interestingly, the selection protocol modifications that we tested herein in which 1 day-old instead of 3 day-old larvae were exposed to dieldrin did not affect this stability.

We showed that the increase in dieldrin concentration and/or exposure time increased female elimination up to a certain threshold. Beyond, a toxic effect of dieldrin was observed on resistant male larvae, reducing male recovery to 50%. Although males are resistant to dieldrin, the insecticide may still cause some aberrations to the nervous system by interfering with ion exchange [19]. In keeping with this hypothesis, we demonstrated that the toxic effect on resistant male larvae was positively correlated with the dieldrin quantity detected in adults. Most interestingly, we showed that when the toxic effect is low, with a male recovery averaging 90%, the dieldrin is hardly quantifiable (using a limit of detection of 2 µg/L corresponding to the quantification sensitivity threshold of the method used herein). Therefore, in a mass production context (growing ~3000 larvae per tray following selection), we detected dieldrin at a concentration of 2 pg/mosquito (always below the quantification threshold) and recorded a male recovery of 92% with a female contamination rate of 4% (1-day-old larvae exposed at 0.4 ppm during 16 h). By comparison, it has been quantified in the GSS of *An*. *Arabiensis* ANO IPCL 1, 9 ng/mosquito with a comparable level of female elimination [16]. We hence detected a thousand times less dieldrin per mosquito.

Although <2 pg per mosquito seems low, it is important to evaluate the amount of dieldrin potentially dispersing in the field with the deployment of a sterile-male program. The abundance of *Ae*. *albopictus* populations on Reunion Island peak at 6000 wild males per hectare during the rainy season [21]. To reach a ratio of 5:1 (sterile male: wild male), approximately 30,000 sterile males have to be released in the field per hectare and week, corresponding to a maximum of 0.06 µg of dieldrin per hectare and week. Thus, a six month treatment corresponds to less than 144 µg of dieldrin per km^2^. The Food and Agriculture Organization/World Health Organization’s acceptable daily intake for the combined total of aldrin and dieldrin for humans is 0.1 µg/kg of body weight, corresponding to 8 µg per day for an 80 kg person [22]. In European legislation, a default value of 10 µg/kg has been adopted to facilitate the control of residues of pesticides for which no maximum residue levels have been established [23]. In humans, the lowest reported lethal dose has been estimated at 5000 µg/kg of body weight [24]. The total dose without negative effects observed is 25 µg/kg and 40 µg/kg of body weight in rats and dogs, respectively [24]. In soils, it has been calculated a predicted no-effect concentration of 0.048 µg/kg [24]. Compared to these data, a level of less than 144 µg of dieldrin per km^2^ for 6 months of releasing sterile males seems negligible. However, the persistence of dieldrin in the field and its bioaccumulation in the food chain were not evaluated herein [16]. Lastly, even if different methods are proposed to degrade dieldrin as bioremediation [25,26], a large volume of liquid wastes generated by mass production can be challenging to recycle. Altogether, presented data show that an efficient sex separation based on dieldrin selection can be achieved with low toxicity on resistant males while dieldrin is hardly quantifiable in adult mosquitoes. Therefore, a cost-benefit analysis of the use of such a GSS should be executed in each environmental context, taking into account local regulations as well as the possibility to treat dieldrin residues in mass production facilities.

## Figures and Tables

**Table 1:** Female contamination rate across generations after dieldrin selection performed on 3-days-old or 1-day-old larvae.

Larvae Stage Exposed	Generations	Number of Male Pupae	Number of Female Pupae	Total of Pupae	Female Contamination (%)
L3	F27	592	7	599	1.17
	F28	306	4	310	1.29
	F29	457	4	461	0.87
	F30	469	3	472	0.64
	F31	2372	15	2387	0.63
	F32	2041	13	2054	0.63
	F33	3331	31	3362	0.92
	F34	2470	16	2486	0.64
L1	F35	2463	10	2473	0.40
	F36	841	7	848	0.83
	F37	1153	8	1161	0.69
	F38	638	5	643	0.78
	F39	3239	26	3265	0.80
	F40	3001	30	3031	0.99
	F41	4326	33	4359	0.76
	F42	4822	37	4859	0.76

**Table 2:** Quantification of dieldrin residues in resistant male adults after insecticide selection applied on 1-day-old larvae for 6 or 24 h. For each exposure time, 1000 1-day-old larvae were exposed to a dieldrin concentration of 0.08 ppm. For each exposure time, all surviving larvae were pooled and reared until pupation. All pupae were manually sex sorted to determine both female contamination rate and male recovery. For each exposure time, 3 pools of 500 adult males each were used for dieldrin quantification.

Exposition Time (h)	Female Contamination (%)	Male Recovery (%)	Dieldrin (µg/L)	Dieldrin (µg/kg)	Dieldrin/Mosquito (µg)
0	45.57	100.00			
6	0.68	90.70	2.05 (±0.07)	18.72 (±1.81)	8.32 × 10^−6^ (±3.08 × 10^−7^)
24	0.86	33.88	486 (±153.48)	3841.09 (±1427.40)	1.94 × 10^−3^ (±6.14 × 10^−4^)

**Table 3:** Quantification of dieldrin residues in resistant male adults after exposure of 1-day-old larvae to different dieldrin concentrations. For each concentration, 160 mg of brushed eggs were allowed to hatch for 8 h in a 30 mL hatching solution. One-day-old larvae were then exposed to dieldrin for 16 h before being rinsed and reared until pupation. All pupae were collected, sex separated and counted. For each repetition, female contamination rate and male recovery were measured. Three repetitions were performed for each dieldrin concentration. Dieldrin quantification was performed on 3 pools of 2000 adult males each for each dieldrin treatment.

Dieldrin Concentration (ppm)	Female Contamination (%)	Male Recovery (%)	Dieldrin (µg/L)	Dieldrin (µg/kg)	Dieldrin/Mosquito (µg)
0	51.1	100			
0.1	18.65 (±3.51)	93.87 (±8.01)	≤2	≤4.71 (±0.03)	≤2 × 10^–6^
0.2	6.87 (±2.78)	87.19 (±5.63)	≤2	≤4.42 (±0.33)	≤2 × 10^–6^
0.4	4.10 (±1.25)	91.89 (±1.17)	≤2	≤4.10 (±0.06)	≤2 × 10^–6^
0.6	3.09 (±0.29)	89.98 (±3.17)	≤2.33	≤4.64 (±1.06)	≤2.33 × 10^–6^
1	2.42 (±1.07)	74.97 (±14.56)	6.33	13.17 (±5.44)	6.66 × 10^–6^

## Data Availability

The data presented in this study are available in “Optimization of dieldrin selection for the genetic sexing of *Aedes albopictus*”.
